# Evaluating Autism Risk Factors and Their Impact on Children in Thi-Qar, Iraq

**DOI:** 10.7759/cureus.72433

**Published:** 2024-10-26

**Authors:** Adnan M Al-Rikabi, Amin T Atya, Naama J Gazar, Mohammed S Almaliki, Huda M Omran

**Affiliations:** 1 Pediatrics, Medical College, University of Thi-Qar, Thi-Qar, IRQ; 2 Pediatrics, Bint Al-Huda Maternity and Children Teaching Hospital ,Thi-Qar Health Directorate, Thi-Qar, IRQ; 3 Pediatrics, American Mission Hospital, Manama, BHR; 4 Genetics: Molecular Genetics, Pulse Health Training Center, Al Jenan Medical Center, Manama, BHR

**Keywords:** autism, children, developmental delays, risk factors, thi-qar

## Abstract

Background

Autism is a neurodevelopmental disorder marked by difficulties in social communication, language, restricted interests, and repetitive behaviors.

Aim

This study aims to identify potential risk factors for autism among children and assess their effects on early developmental skills.

Methods

A case-control study was conducted from September 2022 to September 2023, involving 298 children with autism (265 boys, 33 girls) aged four to 12 from the Thi-Qar Autistic Children Center and private clinics in Iraq. A control group of 300 children (150 boys, 150 girls) was also included. Data were collected through a specialized questionnaire covering demographics, neonatal history (e.g., jaundice, birth asphyxia), parental age, and developmental skills (motor milestones, speech, handwriting).

Results

Among the 298 children with autism enrolled, 265 (89%) were boys and 33 (11%) were girls. Children with autism had a positive family history of autism in 207 (69%) compared to 15 (5%) in the control group (p < 0.001). Paternal age over 35 years at the time of birth was noted in 159 (53%) versus 75 (25%) for the control group (p < 0.001). Maternal age over 35 showed no significant difference (p = 0.23). Hypoxia at birth was present in 153 (51%) children with autism compared to 45 (15%) in the control group (p = 0.001). Significant developmental delays were observed, including speech defects in 210 (70%) children with autism versus 15 (5%) in controls (p < 0.001). In terms of handwriting, 240 (80%) children with autism demonstrated acceptable handwriting skills, while 60 (20%) did not, compared to 264 (88%) in the control group who achieved handwriting skills, resulting in a p-value of 0.23.

Conclusions

The findings indicate that boys are more affected than girls, with significant risk factors including family history, paternal age, and neonatal birth asphyxia. Children with autism demonstrated marked delays in motor skills and speech defects, emphasizing the need for early detection and intervention.

## Introduction

Autism spectrum disorder (ASD) is a neurodevelopmental condition marked by challenges in social communication as well as the presence of repetitive behaviors and restricted interests [1]. These challenges often manifest as difficulties with specialized skills, language development, and limited behavioral patterns within the family context during early childhood [2]. Symptoms typically manifest within the first three years of life and involve three main areas: social interaction, impairment in verbal and non-verbal communication, and repetitive behaviors or restricted patterns of interest [3].

Epidemiological research conducted over the previous fifty years has shown that ASD is becoming more prevalent worldwide. An estimated one in 100 to one in 200 children worldwide are believed to have ASD, with considerable variation between populations mostly attributable to screening, diagnosis, service delivery, and statistical report monitoring [4,5].

Autism is associated with several morphological brain abnormalities, including early brain overgrowth, particularly in gray and white matter, which peaks in early childhood and later stabilizes [6]. Minicolumns, key cortical structures, are often abnormal in size and number, possibly leading to sensory hyperactivation [7]. High-order brain integration regions, such as the frontal cortex, temporal gyri, amygdala, and cerebellum, show structural and functional anomalies, affecting social, emotional, and cognitive processing [1]. Neurochemical imbalances, particularly in glutamate and gamma-aminobutyric acid (GABA) systems, support the hypothesis of disrupted excitation/inhibition regulation in autism [8].

Although the exact etiology of autism is not yet precisely understood, existing scientific literature suggests multifactorial etiopathogenesis. Several potential risk factors have been identified in the literature that may contribute to the development of ASD. These include advanced parental age, prenatal exposure to environmental toxins, maternal infections during pregnancy, and nutritional deficiencies, such as inadequate intake of folic acid and iron [9]. Additionally, genetic predispositions play a significant role, with numerous studies indicating that ASD tends to run in families, suggesting a hereditary component [10].

ASD is likely associated with hundreds of genes that may contribute to its complex inheritance patterns. Key genes such as PTEN, RELN, SHANK3, and neuroligins (NL3, NL4) are involved in neural development and synapse formation, offering insight into the genetic basis of the disorder [11]. Durkin et al.'s work indicated that there is no significant association between an increased risk of ASD and paternal age, even at older ages, or with interpregnancy intervals [12]. The diagnosis of these children is challenging due to the varying presentations of the disorder. Diagnosis is primarily based on behavioral criteria rather than physical examination findings [5,10].

Treatment for autism depends on psychosocial interventions, including structured behavioral, educational, and communication therapies, which are effective for many children. Pharmacotherapy may also help reduce associated symptoms such as anxiety, depression, hyperactivity, and repetitive behaviors [13].

Despite advancements in research on ASD, significant gaps remain in understanding its complexities, especially in Iraq. Current studies often overlook the interplay of genetic, environmental, and neurobiological factors, as well as their interactions with psychosocial aspects and treatment outcomes.

This study aims to fill these gaps by investigating specific neurobiological markers related to ASD, focusing on key variables such as family history, maternal age, and developmental milestones, while also examining neurochemical imbalances in the GABA system. The goal is to enhance early detection and treatment practices for children with ASD in Iraq.

## Materials and methods

A case-control design was conducted in the Al-Nasiriya district of Thi-Qar province, Iraq, from September 2022 to September 2023. The study was conducted at Thi-Qar Center for the Rehabilitation of People with Autism Disorders, located within Bint Alhuda Teaching Hospital for Maternity and Children in Thi-Qar. The case group comprised 298 children diagnosed with ASD, including 265 boys and 35 girls, all aged between four and 12 years. Participants were recruited from the Thi-Qar Autistic Children Center and a private clinic, with diagnoses confirmed by a team of experienced clinicians according to the Diagnostic and Statistical Manual of Mental Disorders (DSM-5) criteria [1] for ASD, which emphasize significant challenges in social communication and interaction, alongside restricted and repetitive behaviors or interests.

The control group consisted of 300 children, matched by age (150 boys and 150 girls), sourced from a nearby primary health center where they received treatment for febrile illnesses. To ensure the study's integrity, inclusion criteria required that participants be within the specified age range, with the case group diagnosed with ASD and the control group confirmed as healthy. Children with comorbid conditions that could interfere with developmental assessments were excluded from the study. Data collection utilized a carefully designed questionnaire that addressed several key areas: demographic details (such as sex and age), neonatal history (including instances of jaundice and birth asphyxia), and developmental milestones (focusing on motor skills like head lag and delayed sitting or walking, as well as speech and handwriting abilities) (Appendix 1). The questionnaire also gathered information on parental age (specifically those aged 35 years and older) and any family history of autism. Interviews were conducted with one or both parents to collect this information, ensuring informed consent was obtained prior to participation. The study received ethical approval from both the Thi-Qar Health Directorate and the Thi-Qar Autistic Children Center. Data analysis was performed using IBM SPSS Statistics for Windows, Version 20 (Released 2011; IBM Corp., Armonk, New York, United States), employing various statistical methods to compare the case and control groups. Chi-square tests were applied to categorical variables, while t-tests were used for continuous variables. Additionally, odds ratios were calculated to assess the strength of associations between risk factors and the outcomes observed. A significance level of p < 0.05 was established to determine statistical significance.

## Results

The study included 598 participants, comprising 298 children diagnosed with ASD and 300 control children. Among the affected children with ASD, 265 (89%) were boys, and 33 (11%) were girls (odd ratio 8.03). In the control group, there were 150 boys (50%) and 150 girls (50%), as shown in Table 1.

**Table 1:** Distribution of participants by age group and gender in the study Statistical test used: chi-square test, significance level: p < 0.05

Group	Age group	Boys	Girls	Total participants	p-value
Case group (ASD)	4 - 6 years	90	10	100	0.001
	7 - 12 years	175	23	198	0.0005
Control group	4 - 6 years	50	50	100	
	7 - 12 years	100	100	200	
Overall Total				598	

Analyzing the risk factors associated with autism revealed several significant associations (Table 2). A notable finding was the strong correlation with family history, where 69% of patients reported a family history of autism compared to only 5% of controls. This yielded an odds ratio (OR) of approximately 43.2, indicating a significant association between family history and autism (P-value: 0.000). In contrast, jaundice showed no significant difference, with 80% of patients and 90% of controls having a history of jaundice, resulting in a P-value of 0.1, indicating that jaundice is not a significant risk factor.

**Table 2:** Risk factors associated with autism in patients compared to controls Statistical test used: chi-square test, significance level: p < 0.05

Risk factors	Patients		Control		P-value
	No.	%	No.	%	
Family history	207	69%	15	5%	0.000
Jaundice	240	80%	270	90%	0.1
Hypoxia	153	51%	45	15%	0.001
Paternal age > 35	159	53%	75	25%	0.001
Maternal age >35	42	14%	36	12 %	0.23

Hypoxia emerged as another significant risk factor, with 51% of patients experiencing hypoxia compared to just 15% of controls. This led to an odds ratio of approximately 5.98, suggesting a significant association with autism risk (P-value: 0.001). Similarly, paternal age greater than 35 years was associated significantly with autism risk, as 53% of patients had fathers older than 35, compared to 25% of controls, yielding an odds ratio of approximately 3.43, indicating a significant association (P-value: 0.001). However, maternal age over 35 did not show a significant association, with 14% of patients and 12% of controls in this category, resulting in a P-value of 0.23.

The evaluation of developmental milestones in the patient group compared to the control group showed several significant differences (Table 3). Delayed head lag was observed in 13% of patients compared to 4% of controls, with a P-value of 0.001, indicating strong statistical significance. Delayed sitting was present in 17% of patients versus 3% of controls, also with a P-value of 0.001. Additionally, 26% of patients experienced delayed walking, compared with only 5% of controls, resulting in a P-value of 0.001.

**Table 3:** Developmental milestones in patients with autism compared to the control group

Milestones	Patients		Control		P-value
	No.	%	No.	%	
Delayed head lag	39	13%	12	4%	0.001
Delayed sitting	51	17%	9	3%	0.001
Delayed walking	78	26%	15	5%	0.001
Speech defect	210	70%	15	5%	0.01
Handwriting	240	80%	264	88%	0.230

The study also highlighted a significant prevalence of speech defects in patients, with 70% affected compared to just 5% in the control group, yielding a P-value of 0.01. Differences in handwriting abilities were not statistically significant, with 80% of patients and 88% of controls able to write, yielding a P-value of 0.230.

## Discussion

The study identified a significant gender disparity among its patient samples, with boys comprising 89% compared to girls at 11% (odd ratio 8.03), as shown in Table 1. These findings, despite the pronounced difference, are consistent with other research in this area, such as McFayden (2021) [14], which reported that 86.4% of their participants were boys. Similarly, Mezzilani et al. (2016) [15] noted a male-to-female prevalence ratio of 4:1. The reasons for this higher prevalence among boys may stem from a combination of social, genetic, and biological factors [16]. This tendency often results in earlier diagnosis of autism in boys than in girls.

This study revealed a significant association between paternal age over 35 years and the occurrence of autism (OR 3.43), with 53% of affected children in this age group compared to 25% in the control group (P = 0.001) (Table 2). These findings align with those of Sasanfar and Hadad (2010) [17], MaGrath and Peteresen (2014) [18], and D'Onofrio and Richert (2014) [19], who observed similar trends. Possible explanations for this association include the exposure of older men to various environmental factors that could affect sperm DNA or the presence of certain autoimmune conditions.

In contrast, our study did not find a significant association between maternal age and autism (14% in the patient group vs. 12% in the control group, P = 0.23), which is consistent with the findings of Reichenberg et al. (2006) [20]. While Lampi et al. (2013) suggested a link between maternal age and autism risk, our results indicate that paternal age may exert a more substantial influence [11]. This difference could be attributed to the narrower childbearing age window for women, reducing their exposure to adverse conditions.

Furthermore, the current study highlighted a significant relationship between neonatal hypoxia and the occurrence of autism, with 51% of affected children experiencing this condition compared to 15% in the control group (Table 2). This finding supports earlier research by Getahun et al. (2013) [21], indicating that neonatal birth asphyxia can increase the risk of certain brain dysplasias. Conversely, our study found no significant link between neonatal hyperbilirubinemia and autism, with 80% of autistic children having neonatal jaundice compared to 90% in the control group. This aligns with Buchmayer et al. (2009) [22], although Lozada et al. (2015) [23] reported a significant association. Such variations may be due to differences in serum bilirubin levels across studies, highlighting the need for further investigation, as elevated bilirubin can potentially disrupt brain development.

Additionally, the study established that the risk of autism significantly increases in families with a previously affected child (69% in the patient group vs. 5% in the control group, P < 0.001). This finding is congruent with research by Lauritsen and Pedersen (2005) [5] and Hansen and Schendel (2019) [10], which also reported a higher risk of autism in families with a history of affected children. These observations reinforce the heritability and genetic basis of autism.

In this study, there was a significant association between delays in early developmental milestones (head lag, sitting, and walking) and the occurrence of autism, with rates of 13%, 17%, and 26%, respectively, compared to 4%, 3%, and 5% in the control group (P < 0.001), as illustrated in Table 3. These results are consistent with Harris (2017), who noted that motor delays could indicate ASD, even as traditional diagnostic criteria focus on social communication deficits and repetitive behaviors [24]. However, Havdahl et al. (2020) [25] reported some differences that were not statistically significant. Autistic children often experience motor delays and atypical postural patterns, underscoring the importance of monitoring developmental milestones closely.

Moreover, the study found a significant correlation between autism and speech defects, with 70% of autistic children exhibiting speech issues compared to only 5% of the control group. This aligns with findings from Romero and Choi (2021) [26], who emphasized that language difficulties are a common feature of autism, albeit with varying severity. Furthermore, Tager-Flusberg and Kasari (2013) found that language skills significantly predict future outcomes for individuals with ASD, including academic success [27].

Finally, our analysis of handwriting revealed no significant differences between autistic children (80% reported acceptable handwriting) and the control group (88% acceptable handwriting, P = 0.23). These findings are somewhat consistent with Fuentes et al. (2009) [28], while Rosenblum and Simhon (2016) observed significantly higher scores in the control group [29]. Additionally, Hellincks and Roeyers (2013) reported that autistic children typically exhibit poorer handwriting skills than their peers [30]. The variability in findings across studies might be attributed to cultural differences or the early introduction of supportive tools in specialized educational environments.

While this study presents valuable insights into autism risk factors, it does have certain limitations. Additionally, the case-control design restricts the ability to establish causality, and the sample was limited to children from specific centers in Thi-Qar, Iraq, potentially affecting the generalizability of the findings to the broader population. Moreover, relying on parental reports for developmental milestones could introduce potential biases. Furthermore, the study did not account for other environmental factors or prenatal exposures that may influence autism risk, which could lead to an incomplete understanding of the conditions contributing to the disorder. Future research should aim to expand the demographic scope and use objective assessments to further validate these results.

## Conclusions

Autism is a pervasive developmental disorder influenced by various risk factors, including a higher prevalence in boys, positive family history, advanced paternal age, and neonatal birth asphyxia, while neonatal jaundice showed no significant association. These findings align with existing literature and highlight adverse outcomes for autistic children, such as developmental delays and speech defects, although handwriting abilities were not significantly affected.

To improve early detection and support, it is essential to implement screening programs, raise awareness among healthcare providers and parents, and conduct further research on risk factors. Additionally, resources for families with a history of autism should be developed, and interventions should focus on enhancing communication and motor skills. Finally, support programs must consider cultural differences to ensure effectiveness and inclusivity.

## Appendices

Appendix 1

Questionnaire for Parents

Study Title: Evaluating Autism Risk Factors and Their Impact on Children in Thi-Qar, Iraq

College of Medicine/Thi-Qar Health Directorate, Iraq

Sheet No: Collecting date: عزيزي ولي الأمر: هل يمكنك تخصيص بعض الدقائق من وقتك الثمين لملء هذه الاستمارة التي ستستخدم في إجراء بحث أجراء بحث أكاديمي حول

التوحد؟

هذا البحث سيساهم في تشخيص حالات التوحد بدقة أعلى في المستقبل. وكن مطمئنًا أن جميع المعلومات ستُعامل بسرية تامة وستستخدم لأغراض البحث فقط.

Dear Parent of a Child with Autism: Could you spare a few minutes of your valuable time to fill out this form, which will be used in conducting an academic research study on autism?

This research will contribute to more accurate diagnosis of autism cases in the future. Rest assured that all information will be treated with complete confidentiality and will be used for research purposes only.

Child’s name ----------------------- Father’s name: ------------------------Father’s cell phone #: ---------------------------Father’s email address: -------------- Mother’s name: ---------------------

Patient Information

Sex: ( ) Male ( ) Female

Date of Birth: ___________________________ Age at Time of Diagnosis: ___________________________ Age at Time of Study: ___________________________ Date of Information Recording: ___________________________ Address: ___________________________

Parental Information

() Mother's Age at Childbirth: () Father's Age at Childbirth:                                                               

Birth History

Delivery Method:

() Normal Vaginal Delivery () Elective C/S () Emergency C/S

History of Difficult Labor:

()Yes ()No

Birth Asphyxia:

()Yes ()No

Admission to NICU:

()Yes ()No

History of Neonatal Jaundice:

()Yes ()No

Treatment or Admission to NICU for Jaundice:

()Yes ()No

Family History

Previous Autistic Child: ( ) One Child ( ) Two Children ( ) More than Two Children ()None

Developmental Milestones

()Head Control and Rolling: Age ____ ()Sitting Without Support: Age ____ ()Walking Without Support: Age ____ ()First Word Spoken: Age ____ ()Speech Understood: Age ____ ()Speaking in Sentence: Age ____ ()Speaking Fluently: Age ____

Handwriting and Speech Processing No Handwriting:

()Yes ()No

Ineffective Writing:

()Yes ()No

Defect in Word Processing:

()Yes

()No

ِEffective Writing:

()Yes ()No

Parental Consent

Parental Consent for Child to be Enrolled in Research Study: ( ) I consent to my child participating in this research study. ( ) I do not consent to my child participating in this research study.

Additional Comments: *Please provide any additional information or comments regarding your child's development:*

__________________________________________________________________________ __________________________________________________________________________

Parent’s Signature: _________________________________ Date Signed: _______________

Thank You! Thank you for participating in this study. Your responses are valuable for understanding autism risk factors.
